# Antibiotic Resistance-Susceptibility Profiles of *Enterococcus faecalis* and *Streptococcus* spp. From the Human Vagina, and Genome Analysis of the Genetic Basis of Intrinsic and Acquired Resistances

**DOI:** 10.3389/fmicb.2020.01438

**Published:** 2020-06-26

**Authors:** Auttawit Sirichoat, Ana Belén Flórez, Lucía Vázquez, Pranom Buppasiri, Marutpong Panya, Viraphong Lulitanond, Baltasar Mayo

**Affiliations:** ^1^Departamento de Microbiología y Bioquímica, Instituto de Productos Lácteos de Asturias (IPLA-CSIC), Villaviciosa, Spain; ^2^Department of Microbiology, Research and Diagnostic Center for Emerging Infectious Diseases (RCEID), Faculty of Medicine, Khon Kaen University, Khon Kaen, Thailand; ^3^Instituto de Investigación Sanitaria del Principado de Asturias (IISPA), Oviedo, Spain; ^4^Department of Obstetrics and Gynecology, Faculty of Medicine, Khon Kaen University, Khon Kaen, Thailand; ^5^College of Medicine and Public Health, Ubon Ratchathani University, Ubon Ratchathani, Thailand

**Keywords:** antibiotic resistance, tetracycline resistance, genome analysis, vaginal strains, *Enterococcus faecalis*, *Streptococcus anginosus*, *Streptococcus salivarius*

## Abstract

The spread of antibiotic resistance is a major public health concern worldwide. Commensal bacteria from the human genitourinary tract can act as reservoirs of resistance genes playing a role in their transfer to pathogens. In this study, the minimum inhibitory concentration of 16 antibiotics to 15 isolates from the human vagina, identified as *Enterococcus faecalis*, *Streptococcus anginosus*, and *Streptococcus salivarius*, was determined. Eight isolates were considered resistant to tetracycline, five to clindamycin and quinupristin-dalfopristin, and four to rifampicin. To investigate the presence of antimicrobial resistance genes, PCR analysis was performed in all isolates, and five were subjected to whole-genome sequencing analysis. PCR reactions identified *tet*(M) in all tetracycline-resistant *E. faecalis* isolates, while both *tet*(M) and *tet*(L) were found in tetracycline-resistant *S. anginosus* isolates. The *tet*(M) gene in *E. faecalis* VA02-2 was carried within an entire copy of the transposon Tn*916*. In *S. anginosus* VA01-10AN and VA01-14AN, the *tet*(M) and *tet*(L) genes were found contiguous with one another and flanked by genes encoding DNA mobilization and plasmid replication proteins. Amplification and sequencing suggested the *lsaA* gene to be complete in all *E. faecalis* isolates resistant to clindamycin and quinupristin-dalfopristin, while the gene contain mutations rendering to a non-functional LsaA in susceptible isolates. These results were subsequently confirmed by genome analysis of clindamycin and quinupristin-dalfopristin resistant and susceptible *E. faecalis* strains. Although a clinical breakpoint to kanamycin for *S. salivarius* has yet to be established, *S. salivarius* VA08-2AN showed an MIC to this antibiotic of 128 μg mL^–1^. However, genes involved in kanamycin resistance were not identified. Under the assayed conditions, neither *tet*(L) nor *tet*(M) from either *E. faecalis* or *S. anginosus* was transferred by conjugation to recipient strains of *E. faecalis*, *Lactococcus lactis*, or *Lactobacillus plantarum*. Nonetheless, the *tet*(L) gene from *S. anginosus* VA01-10AN was amplified by PCR, and cloned and expressed in *Escherichia coli*, to which it provided a resistance of 48–64 μg mL^–1^ to tetracycline. Our results expand the knowledge of the antibiotic resistance-susceptibility profiles of vaginal bacteria and provide the genetic basis of their intrinsic and acquired resistance.

## Introduction

The indiscriminate use of antibiotics in the treatment of infections and as prophylactic agents has potentiated the emergence and spread of different types of antibiotic resistance (Bengtsson-Palme et al., 2018). Antibiotic-resistant bacteria pose an increasingly important public health challenge worldwide. When resistance to an antibiotic is inherent to all strains of a bacterial species, it is generally referred to as intrinsic resistance. In contrast, when a strain of a typically susceptible species is resistant to a given antibiotic, it is considered to be acquired resistance (EFSA FEEDAP Panel, 2012). A bacterial species may have intrinsic resistance to an antibiotic due to the lack of target, the possession of low-affinity targets, cell impermeability to the antibiotic, or the existence of multidrug efflux mechanisms that excrete it (Cox and Wright, 2013). The acquisition of resistance occurs via genetic mutation or by antimicrobial resistance (AMR) gene gain via horizontal transfer. Transference is fueled by the presence of AMR genes in conjugative and mobilizable genetic elements, such as plasmids, transposons, or integrative and conjugative elements (van Hoek et al., 2011). Currently, there is a pressing need to limit the spread of such genes, which could be transferred to opportunistic and pathogenic bacteria (Peterson and Kaur, 2018).

The vagina represents a complex ecosystem in which many microbial species occur in varying numbers and proportions (Huang et al., 2014). Microorganisms exist in a finely tuned mutualistic relationship with the host, and provide the first line of defense against colonization and infection by pathogens (Smith and Ravel, 2017). The dominant bacterial species in human vagina belong to the genus *Lactobacillus* (Martin, 2012; Sirichoat et al., 2018). However, species of other genera, such as *Gardnerella, Atopobium, Prevotella, Corynebacterium, Anaerococcus, Peptoniphilus*, can, under certain conditions, constitute majority populations (Martin, 2012; Sirichoat et al., 2018). Species of *Enterococcus* and *Streptococcus* genera are frequently isolated as subdominant populations. Lactic acid-producing strains of these biotypes might possibly be used as vaginal probiotics to prevent or treat vaginal infections by inhibiting the development of pathogens (Nami et al., 2014). However, some strains of these genera are also known to be opportunistic pathogens causing occasional disease (Krzyściak et al., 2013; Ben Braïek and Smaoui, 2019). Further, enterococci are well-known reservoirs of antibiotic resistance genes, as they harbor abundant conjugative plasmids and transposons that might be broadly transferred to other bacteria (Miller et al., 2014). The intra- and inter-genus transfer of AMR genes from and to streptococci has been documented as well (Chajêcka-Wierzchowska et al., 2019). Indeed, it is known that the transfer of AMR genes between bacterial species does occur in the vagina (Nogacka et al., 2017). It is for these reasons that enterococci and streptococci -with the exception of *Streptococcus thermophilus*- have been refused Generally Regarded as Safety (GRAS) status by the US Food and Drug Administration (FDA, 2010), and are not recommended for the Qualified Presumption of Safety (QPS) by the European Food Safety Authority (EFSA) (EFSA BIOHAZ Panel, 2017).

While a number of studies have been conducted on antibiotic resistance in pathogens such as *Gardnerella vaginalis*, group B streptococci (Nagaraja, 2008; Bolukaoto et al., 2015), and lactic acid bacteria (LAB) from the human vagina (Martín et al., 2008; Fuochi et al., 2019), the antibiotic resistance-susceptibility profiles of other vaginal-dwelling cocci have yet to be determined. The aim of the present work was to examine the antibiotic resistance/susceptibility profiles of a set of cocci isolates recovered from the vagina of healthy Thai women, and to investigate by PCR and whole genome sequencing and analysis the genetic basis of the intrinsic and acquired resistances identified. The capacity of some resistances to undergo horizontal transfer *in vitro* was also assessed.

## Materials and Methods

### Selection of Volunteers and Sample Collection

This study was approved by the Khon Kaen Ethics Committee in Human Research (Ref. HE581191). The women of this study were selected among patients attending the gynecological clinic of Srinagarind Hospital, Faculty of Medicine, Khon Kaen University (Khon Kaen, Thailand). To be eligible, women had to be 20–45 years old, not be pregnant, have regular menstruation, have no serious underlying disease (e.g., diabetes mellitus or systemic lupus erythematous) and no clinical genitourinary symptoms on examination, and had no antibiotic treatment during the last 6 months. Before sampling, volunteers signed a written informed consent. Vaginal exudates were taken by swabbing the lateral, anterior and posterior vaginal walls with sterile cotton-tipped applicators (performed at the clinic); these were then placed in sterile recipients containing reduced Amies transport medium (Oxoid, Basingstoke, United Kingdom) and stored at 4°C until culturing later on the same day.

### Isolation of Bacteria

Vaginal swabs were suspended and serially diluted in de Man, Rogosa and Sharpe (MRS) broth (Oxoid), and the dilutions spread on MRS agar plates containing 0.5% (w/v) CaCO_3_ (BDH, Poole, United Kingdom). Plates were incubated at 37°C for 48 h in aerobic or anaerobic (with Anaerocult; Merck, Darmstadt, Germany) conditions. Individual colonies surrounded by clear halos from both culture conditions were randomly selected and subcultured in MRS and incubated under the same conditions. Isolates were then screened by Gram staining, colony morphology, and the catalase test. Their hemolytic activity was assessed using sheep blood agar plates (Oxoid). Only Gram-positive, catalase negative, and γ-hemolytic cocci were selected. These isolates were routinely cultivated in MRS agar and incubated at 37°C for 48 h, and then stored in MRS broth supplemented with 15% (w/v) glycerol (Merck) at −80°C.

### Identification of Isolates

Genomic DNA extraction and purification from the isolates was performed using the GenElute Bacterial Genomic DNA kit (Sigma-Aldrich, St. Louis, Mo., United States) following the manufacturer’s instructions. Purified DNA was used as a template for amplifying the 16S rRNA genes using the universal primers 27F (5′-AGAGTTTGATCCTGGCTCAG-3′) and 1492R (5′-GGTTACCTTGTTACGACTT-3′) (Frank et al., 2008) and Taq polymerase (Ampliqon, Odense, Denmark). The PCR conditions were as follows; one cycle of 95°C for 5 min, followed by 35 cycles of denaturation at 94°C for 30 s, primer annealing at 55°C for 45 s, and extension at 72°C for 2 min. A final extension step at 72°C for 10 min was then performed. Amplified products were checked by electrophoresis using 1% agarose gels, visualized after 90 min, and photographed under UV light using a G Box equipment (SynGene, Cambridge, United Kingdm). The amplified products were purified using a GenElute PCR Clean-Up Kit (Sigma-Aldrich) column, and subjected to standard Sanger DNA sequencing at Macrogen (Madrid, Spain). Strains were identified at the species level by comparing their sequences to those in the NCBI database using the BLAST tool (Altschul et al., 1997). Sequences sharing 97% identity or higher were deemed to belong to the same species.

### Molecular Typing of Isolates

Isolates were typed using random amplified polymorphic DNA-PCR (RAPD-PCR) and repetitive element-PCR (rep-PCR) fingerprinting methods, using primer M13 (5′-GAGGGTGGCGGTTCT-3′) as described by Rossetti and Giraffa (2005), primer BoxA2R (5′-ACGTGGTTTGAAGAGATTTTCG-3′) as described by Koeuth et al. (1995), and primer OPA18 (5′-AGGTGACCGT-3′) as described by Mättö et al. (2004). PCR reaction mixtures contained 2 μL of each purified genomic DNA (≈100 ng), 12.5 μL of 2 × Master Mix RED (Ampliqon), 5 μL of either primer (10 μM) and molecular biology grade water (Sigma-Aldrich) in a total volume of 25 μL. The PCR conditions were as follows; one cycle of 95°C for 7 min, followed by 40 cycles of denaturation at 95°C for 30 s, primer annealing at 42°C (M13), 40°C (BoxA2R) or 32°C (OPA18) for 1 min, and extension at 72°C for 4 min. A final extension step at 72°C for 10 min was then performed. Amplification products were separated by electrophoresis using 2.5% agarose gel and visualized as above. Pattern profiles were clustered and compared using the unweighted pair group method with arithmetic mean (UPGMA), and their similarity expressed via the simple matching (SM) coefficient in GeneTools v.4.03 (SynGene). Triplicate analysis by RAPD-PCR and rep-PCR techniques with all three primers identified a combined repeatability of the fingerprinting of 94%; consequently, profiles with <94% similarity were considered different strains.

### Antibiotic Susceptibility Testing

The minimum inhibitory concentration (MIC) of 16 antibiotics was determined according to ISO standard 10932:2010 (IDF, 2010) using VetMIC plates (National Veterinary Institute of Sweden, Uppsala, Sweden). The plates contained twofold serial dilutions of the antibiotics gentamicin, kanamycin, streptomycin, neomycin, tetracycline, erythromycin, clindamycin, chloramphenicol, ampicillin, penicillin, vancomycin, quinupristin-dalfopristin, linezolid, trimethoprim, ciprofloxacin, or rifampicin. Individual colonies grown on Muller-Hinton agar plates (Oxoid) were suspended in 2 mL sterile saline (0.9% NaCl solution) to obtain a density corresponding to McFarland standard 1 (≈3 × 10^8^ cfu mL^–1^). This suspension was further diluted 1:1000 in Muller-Hinton broth to achieve a final cell concentration of approximately 3 × 10^5^ cfu mL^–1^. One-hundred microliters of this inoculum were then added to each well of the VetMIC plates. Following incubation for 48 h at 37°C, MICs were visually determined as the lowest antibiotic concentration at which growth was inhibited. The concentration range for clindamycin in the VetMIC plates was insufficient to determine the actual MIC for some isolates; this was determined using the MICE system (Oxoid) following the manufacturer’s recommendations. A strain was considered phenotypically resistant to an antibiotic when it was not inhibited by a concentration higher than the established clinical breakpoint retrieved from the CLSI guidelines (CLSI, 2019). When not covered, the microbiological cut-off values of the European Committee on Antimicrobial Susceptibility Testing (EUCAST) (EUCAST, 2019) were adopted.

### PCR Detection of Antibiotic Resistance Genes

Genes involved in tetracycline resistance were searched for by PCR using the degenerate primer pairs DI-DII and Tet1-Tet2 targeting genes encoding ribosomal protection proteins (RPPs). Specific primers were used for the detection of genes coding for resistance to tetracycline [*tet*(M), *tet*(O), *tet*(S), *tet*(W), *tet*(K), and *tet*(L)], erythromycin and clindamycin [*erm*(A), *erm*(B), *erm*(C), *erm*(F), and *mef*(A)], chloramphenicol (*cat*), β-lactam antibiotics (*bla*), aminoglycosides [*aac(6’)-aph(2″)* and *aad*(E)], clindamycin (*lsaA*), and vancomycin (*vanA*) (Supplementary Table 1). The reaction mixtures (50 μL) contained 25 μL of Taq 2 × Master Mix RED, 1.5 μL of each primer (10 μM), 2 μL of each purified genomic DNA (≈100 ng) and 20 μL of molecular biology grade water. The PCR conditions were as follows; initial denaturation at 94°C for 5 min, 35 cycles of 94°C for 1 min, an appropriate annealing temperature (Supplementary Table 1) for 1 min, 72°C for 2 min, and a final extension step at 72°C for 10 min. The amplified products were then electrophoresed, visualized, and recorded. Selected amplicons were purified and sequenced, and their sequences compared as above.

### Genome Sequencing, Annotation, and Analysis

A library of 0.5 kbp was constructed from the total genomic DNA belonging to five vaginal *E. faecalis* and *Streptococcus* spp. strains, and paired-end sequenced using a Genome Sequencer Illumina HiSeq 1000 System at Eurofins Genomics (Konstanz, Germany). Quality-filtered reads were assembled in contigs using Spades v.3.6.2 software (Bankevich et al., 2012). Genomes were annotated using the RAST annotation system (Aziz et al., 2008) and the NCBI Prokaryotic Genome Annotation Pipeline (Tatusova et al., 2016). DNA and deduced protein sequences of interest were examined for homology using the on-line BLAST tool as above. The homology of genes and proteins involved in antimicrobial resistance was further investigated by searching the CARD (McArthur et al., 2013), ResFinder (Zankari et al., 2012), and ARG-ANNOT (Gupta et al., 2014) databases.

### Conjugation Experiments

Strains harboring *tet*(M) or both *tet*(M) and *tet*(L) were selected for filter mating conjugations, using as recipient strains *Lactococcus lactis* subsp. *cremoris* MG1614, *L. lactis* subsp. *lactis* biovar. *diacetylactis* Bu2-60, *E. faecalis* OG1RF and *Lactobacillus plantarum* NC8. All recipients were plasmid-free and susceptible to tetracycline. The two *L. lactis* strains were resistant to streptomycin (500 μg mL^–1^) and rifampicin (100 μg mL^–1^), *E. faecalis* OG1RF was resistant to rifampicin (100 μg mL^–1^) and fusidic acid (25 μg mL^–1^), and *L. plantarum* NC8 was resistant to streptomycin (500 μg mL^–1^).

Conjugation was performed by filter mating as previously described (Flórez et al., 2008) with minor modifications. Briefly, donor and recipient strains were grown separately overnight in M17 broth (Formedium, Hunstanton, United Kingdom) with 0.5% glucose (w/v) (GM17) at 32°C (*L. lactis*) or 37°C (*E. faecalis*, *S. anginosus*, and *L. plantarum*). After incubation, the donor and recipient strains were mixed (10:1) and filtered through a sterile membrane filter with a pore size of 0.45 μm (PALL, Life Sciences, Mexico). The filters were then incubated on GM17 agar with and without 50 ng per mL of tetracycline for 24 h at the optimal temperature for the recipient. After incubation the filters were washed with 2 mL of GM17 broth and suspended in the same medium. Finally, serial dilutions of the suspension were plated on GM17 agar with appropriate antibiotics for counting donor and recipient numbers, and selecting transconjugants. The plates were incubated at 32–37°C for 24–72 h and, to distinguish transconjugants from mutants, the colonies were typed with primer OPA18 using the RAPD-PCR technique as described above.

### DNA Manipulation and Cloning

The general procedures used for DNA manipulation were essentially those described by Sambrook and Russell (2001). Restriction endonucleases (Fermentas, St. Leon-Rot, Germany), T4 DNA ligase (Roche, Mannheim, Germany), and Pfx DNA polymerase (Invitrogen, Carlsbad, CA, United States) were used according to the manufacturers’ instructions. The *tet*(L) gene from *S. anginosus* VA01-10AN was amplified by PCR with primers incorporating *Sal*I and *Eco*RI restriction enzyme sites (Supplementary Table 1). Amplicons and pUC19 were digested with the two enzymes, ligated and electroporated into electrocompetent *Escherichia coli* DH10B cells using a GenePulser apparatus (Bio-Rad Laboratories, Richmond, CA, United States). Plasmid DNA from *E. coli* was purified as described by Sambrook and Russell (2001), analyzed by restriction enzyme analysis and sequenced.

### GenBank Accession Numbers

The genome sequences of *E. faecalis* VA02-2 and VA37-4, *S. anginosus* VA01-10AN and VA01-14AN, and *S. salivarius* VA08-2AN were deposited in the GenBank database under the BioProject PRJNA604905 with biosample accession numbers SAMN14086434, SAMN14086702, SAMN14086703, SAMN14086908, and SAMN14086913, respectively.

## Results

### Isolation and Identification of Vaginal Cocci

Fifteen vaginal cocci colonies surrounded by clear halos on MRS agar with 0.5% CaCO_3_ (thus producing large quantities of lactic acid) were recovered. All isolates were Gram-positive, catalase negative, and γ-hemolytic. Sequencing and sequence comparison of the 16S rRNA genes identified the isolates as *Enterococcus faecalis* (nine isolates), *Streptococcus anginosus* (four isolates), and *Streptococcus salivarius* (two isolates) (Table 1).

**TABLE 1:** Minimum inhibitory concentration (MIC) values of 16 antibiotics to vaginal *Enterococcus faecalis* and *Streptococcus* spp. isolates.

Species	Strain	Antibiotic^a^ (MIC as μg/mL)															
		GEN	KAN	STR	NEO	TET	ERY	CLI	CHL	AMP	PEN	VAN	QDA	LIN	TMP	CIP	RIF
*E. faecalis*	VA01-17	8	32	128	64	32	2	0.25	8	0.5	2	1	1	2	1	1	4
	**VA02-2**	8	128	128	32	32	2	0.25	8	1	2	1	0.5	2	1	2	8
	VA32-9	8	64	128	64	16	4	0.5	8	0.5	2	1	0.5	2	0.5	2	8
	VA01-13	8	64	128	32	16	4	0.5	8	0.5	2	2	0.5	2	0.5	1	8
	VA02-1	16	64	128	128	1	4	256	16	0.5	2	1	>8	4	0.5	2	8
	VA16-2AN^b^	16	64	128	256	1	4	256	16	0.5	2	1	8	4	0.5	2	4
	VA36-16AN	16	64	64	64	1	2	256	16	1	2	1	8	4	0.5	2	4
	**VA37**-**4**	16	64	128	128	0.5	2	256	8	0.5	2	4	>8	2	0.25	2	2
	VA24-3AN	16	64	128	128	1	4	256	16	0.5	2	1	8	4	0.5	2	2
**Breakpoint (μg/mL)^c^**		**128**	**1,024**	**512**	–	**8**	**8**	**4**	**32**	**8**	**16**	**4**	**4**	**8**	-	**4**	**4**
*S. anginosus*	VA01-9AN	8	64	32	32	32	0.06	0.12	2	0.25	0.12	1	1	1	≤0.12	32	0.25
	VA01-20	4	64	32	32	32	0.06	0.12	4	0.25	0.12	1	2	1	≤0.12	64	0.5
	**VA01**-**10AN**	2	32	8	16	32	0.06	0.12	2	0.25	0.12	1	1	0.5	0.25	64	0.25
	**VA01**-**14AN**	1	8	4	8	16	0.06	0.25	2	0.25	0.12	1	1	1	0.25	32	1
*S. salivarius*	**VA08**-**2AN**	2	128	16	8	2	0.12	0.12	2	1	0.5	1	1	1	8	4	0.5
	VA08-1AN	≤0.5	16	8	1	0.25	0.03	≤0.03	2	0.5	0.5	0.5	0.5	0.5	8	2	≤0.12
**Breakpoint (μg/mL)^c^**		**128**	–	**64**	–	**8**	**1**	**1**	**16**	**8**	**4**	**2**	**4**	–	–	–	–

*^*a*^GEN, gentamicin; KAN, kanamycin; STR, streptomycin; NEO, neomycin; TET, tetracycline; ERY, erythromycin; CLI, clindamycin; CHL, chloramphenicol; AMP, ampicillin; PEN, penicillin; VAN, vancomycin; QDA, quinupristin-dalfopristin; LIN, linezolid; TMP, trimethoprim; CIP, ciprofloxacin; RIF, rifampicin. ^*b*^Strains coded with AN were recovered from plates cultured in anaerobiosis. In bold, strains subjected to whole genome sequencing. ^*c*^Breakpoint values were set by compiling data from CLSI (2019) and EUCAST (2019). Gray-shaded boxes show MIC values higher than the breakpoints or that deviate from MIC values distribution. –, breakpoint not established.*

### Antibiotic Susceptibility Patterns

Since the number of isolates was small, and since large phenotypic variation has been seen even among closely related isolates (Kürekci et al., 2016), all were subjected to susceptibility testing. According to the compiled clinical breakpoints from CLSI (2019) and EUCAST (2019) (Table 1), all isolates were susceptible or showed low MIC values to gentamicin, streptomycin, erythromycin, chloramphenicol, ampicillin, penicillin, vancomycin, linezolid, and trimethoprim. Among the *E. faecalis* isolates, four showed an MIC of tetracycline higher than the breakpoint (8 μg mL^–1^). Five *E. faecalis* isolates were considered resistant to both clindamycin and quinupristin-dalfopristin (MICs > 256 and ≥ 8 μg mL^–1^, respectively), and four to rifampicin (MIC 8 μg mL^–1^). The MIC values of *E. faecalis* isolates to neomycin (32–128 μg mL^–1^) were comparable to those seen for all other aminoglycosides. As for the *Streptococcus* spp., all four *S. anginosus* isolates showed resistance to tetracycline. Although a breakpoint for ciprofloxacin in *S. anginosus* has yet to be established, the MICs of this antibiotic to isolates of this species (32–64 μg mL^–1^) was higher than in all others. Finally, the MIC of kanamycin to *S. salivarius* VAN08-2AN was three dilutions higher than that to the other isolate of this species.

### PCR Detection of Antibiotic Resistance Genes

As for the phenotypic analysis, the small number allowed us to test by PCR the presence of a vast array of antibiotic resistance genes in all isolates; this will uncover the possible existence of the so-called silent genes (Flórez et al., 2006). Amplification of genes involved in tetracycline resistance was done with universal primer for genes encoding RPPs, as well as with gene-specific primers. Using universal primers, amplification was obtained for all eight tetracycline resistant isolates (Table 1). Further analysis using *tet*(M)-specific primers showed amplicons to be produced for all eight isolates. In addition, an amplicon was produced for all tetracycline-resistant *S. anginosus* with *tet*(L)-specific primers. The sequenced amplicons of *tet*(M) were nucleotide-identical among themselves. In contrast, those of *tet*(L) from *S. anginosus* showed identity to different gene variants.

No silent or inactive genes associated with resistance to chloramphenicol (*cat*), β-lactam antibiotics (*bla*), aminoglycosides [*aac*(6*’*)-*aph*(2*″*), and *aad*(E)], glycopeptides [*van*(A)], macrolides [*erm*(A), *erm*(B), *erm*(C), *erm*(F), and *mef*(A)], or encoding ribosomal protecting proteins [*tet*(O), *tet*(S), and *tet*(W)], or efflux protein [*tet*(K)] involved in tetracycline resistance, were detected in any of the isolates.

The putative housekeeping efflux-encoding gene *lsaA* of *E. faecalis* was amplified from both clindamycin and quinupristin-dalfopristin resistant and susceptible strains, and their sequences compared. The *lsaA* gene was complete in strains showing resistance, while mutations generating stop codons were noted in sequences from all strains displaying susceptibility to these antibiotics. As a high-fidelity polymerase with proofreading activity was not used, these results, however, cannot be considered conclusive.

### Genome Sequence and Analysis of Antibiotic Resistant Strains

The pooled results of the independent fingerprinting analyses of the isolates by RAPD-PCR and rep-PCR suggested the presence of six strains among the *E. faecalis* isolates, two among the *S. anginosus*, and one among the two *S. salivarius* isolates (Supplementary Figure 1). Based on the phenotypic and genotypic results, five strains were selected for genome sequencing: *E. faecalis* VA02-2 (resistant to tetracycline and rifampicin), *E. faecalis* VA37-4 (resistant to clindamycin and quinupristin-dalfopristin), and *S. anginosus* VA01-10AN and 01-14AN (resistant to tetracycline, and MIC of ciprofloxacin 64 and 32 μg mL^–1^, respectively), and *S. salivarius* VA08-2AN (MIC of kanamycin 128 μg mL^–1^).

As expected, the genome analysis confirmed the presence of *tet*(M) and *tet*(L) in the tetracycline-resistant strains in which they were previously detected by PCR. These genes were detected by searches in all three specific antimicrobial resistance databases (CARD, ResFinder, and ARG-ANNOT). CARD and ARG-ANNOT databases further identified wild type genes *dfrE* (encoding a dihydrofolate reductase) and *efrA* (encoding an ATP-binding cassette-ABC efflux pump) as involved in antibiotic resistance in *E. faecalis* (García-Solache and Rice, 2019). Mutations in these genes have been associated with resistance to trimethoprim, and macrolide and fluoroquinolone resistance, respectively. In addition, ResFinder and ARG-ANNOT also included *lsa*A gene from *E. faecalis* as an antibiotic resistance gene.

**FIGURE 1:**
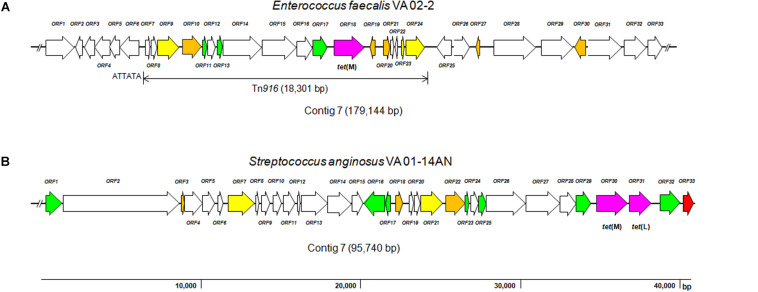
Diagram showing the genetic organization of ORFs in the contigs around the tetracycline resistance genes identified in the genome sequence of *Enterococcus faecalis* VA02-2 (**A**; JAAJBI000000007.1) and *Streptococcus anginosus* VA01-14AN (**B**; JAAJBF000000008.1). Color code of the different ORFs: purple, antibiotic resistance genes, *tet*(M) of *E. feacalis* (locus tag G5T25_09190) and *tet*(M) and *tet*(L) of *S. anginosus* (locus tags G5T15_06645 and G5T15_06650, respectively); red, genes involved in plasmid replication and control; yellow, transposase- associated genes; green, integrase-, mobilization-, and conjugation-associated genes; orange, genes encoding transcription regulators; white, genes involved in other processes. The broken line symbol indicates that the sequence of the contig extends beyond this point.

The *tet*(M) gene in *E. faecalis* VA02-2 was found to be encoded on a large contig of 179,144 bp (JAAJBI010000007.1; locus tag G5T25_09190). The genetic organization of around 40 kbp of this contig is depicted in Figure 1A. A detailed analysis of all open reading frames (ORFs) in this section of the contig is summarized in Supplementary Table 2. *tet*(M) was identified within a DNA region harboring a complete copy of the transposon Tn*916* (18,031 bp). Compared to the Tn*916* copy in *E. faecalis* DS16 (U09422.1), the copy in VA02-2 contains 14 nucleotide replacements, two deletions of one base pair, and an insertion of one base pair. None of these changes had any effect on translation, except for one of the insertions which induced a shorter version of the protein encoded by locus tag G5T25_09170 (ORF15; Supplementary Table 2). The sequence homology to that of ORF15 of Tn*916* in *E. faecalis* DS16 is lacking from amino acid 653 onward. This gene encodes a functionally uncharacterized protein containing a YtxH domain. Nonetheless, versions of Tn*916* with identical nucleotide and amino acid sequences to those of *E. faecalis* VA02-2 can be found for many strains in databases, including *E. faecalis* KUB3007 (AP018543.1), *Enterococcus durans* VREdu (CP042597.1), *Staphylococcus aureus* B6-55A (CP042110.1), and *Streptococcus agalactiae* PLGBS13 (CP029749.1). Tn*916* in *E. faecalis* VA02-2 appears to be inserted into the intergenic region between the genes encoding an ATP-binding subunit of an ABC transporter for betaine/proline/choline (ORF6; locus tag G5T25_09120) and a glyoxalase (ORF25; locus tag G5T25_09225) (Figure 1A and Supplementary Table 2). In the downstream region, the nucleotide sequence of Tn*916* is immediately followed by chromosomal sequences of *E. faecalis*, while in the upstream region a scar of six base pairs (ATTATA) between the transposon and the chromosome seems to be inserted (Figure 1A).

Similarly to *tet*(M) in *E. faecalis*, the *tet*(M) and *tet*(L) genes in *S. anginosus* VA01-10AN (JAAJBG010000010.1) and VA01-14AN (JAAJBF000000008.1) were identified as being adjacent in contigs larger of 65.0 kbp with an identical genetic organization. This result reinforces the genetic relatedness of the strains as determined by the typing analyses (Supplementary Figure 1). In contrast to *E. faecalis* VA02-2 that proved to be plasmid-free, a plasmid of 17.5 kbp was present in both *S. anginosus* VA01-10AN and VA01-14AN strains (data not shown). However, *tet*(M) and *tet*(L) were not found to be associated with this plasmid. The genetic organization around the *tet*(M) and *tet*(L) gene in *S. anginosus* VA01-14AN is depicted in Figure 1B, and the analysis of ORFs in this region is outlined in Supplementary Table 2. The two resistance genes were found to be arranged in tandem, separated by an intergenic region of only 102 bp. Canonical ribosome binding sites (GGAGG) were identified in front of the two genes, as well as upstream sequences that might function as promoters. The genes were flanked by ORFs encoding a truncated protein involved in the replication of plasmid pUB110 (from *Staphylococcus aureus*) on one side (locus tag G5T15_06655), and by Tn*916*-related ORFs involved in DNA mobilization on the other (locus tag G5T15_06635). The same organization of the region harboring the *tet*(M) and *tet*(L) is found in plasmids, e.g., in pC27-2 of *Enterococcus faecium* (MH784602.1), and in the chromosome of strains belonging to different species, such as *Streptococcus pasteurianus* ATCC 43144 (AP012054.1), *Streptococcus equinus* NCTC11436 (LR134203.1), *Streptococcus pneumoniae* IC161 (HG799499.1), *E. faecium* E4413 (LR135185.1), *E. faecalis* ATCC 29212 (CP008816.1), and many others. The existence of mobilization features from transposons and plasmids around the genes resembles somehow the genetic organization of the integrative and conjugative elements (ICEs) of staphylococci (Sansevere and Robinson, 2017).

Regarding the genetic basis of other resistances, the *lsaA* gene (JAAJBH010000005.1; locus tag G5T22_RS09575) of *E. faecalis* VA37-4 (clindamycin and quinupristin-dalfopristin resistant) was identified in a contig of 249,478 bp. This gene is believed to encode an ABC superfamily efflux protein, which provides resistance to lincosamides, pleuromutilins, and streptogramin A antibiotics (Singh et al., 2002). The gene and its derived protein sequence were analyzed and compared to those from susceptible and resistant strains of the same species. The *lsaA* gene of VA37-4 was found to be complete, encoding a protein of 498 amino acids. The LsaA of VA37-4 was of the same length and showed only two amino acid replacements (at positions 77 and 495) to the protein of *E. faecalis* OG1RF (NC_017316.1), another clindamycin and quinupristin-dalfopristin resistant strain (Supplementary Figure 2). Conversely, the *lsaA* gene sequence in the susceptible strain *E. faecalis* VA02-2 contained two mutations that destroyed the ORF and lead to the production of a truncated peptide of 152 amino acids long (Supplementary Figure 2). Thus, genome analysis confirmed the previous *lsaA* amplification and sequencing results in both *E. faecalis* VA02-2 and VA37-4. As concerns rifampicin resistance, genome analysis of VA02-2 strain failed to identify the genetic basis of its MIC to rifampicin (8 μg mL^–1^). Genes and well-described mutations (such as those in the *rpoB* gene) known to be involved in rifampicin resistance were not detected in this strain. Genome analysis of *S. salivarius* VA08-2AN did not identify either any gene known to be involved in kanamycin and/or aminoglycoside resistance (MIC 128 μg mL^–1^). Indeed, mutations in key genes known to cause resistance to aminoglycosides, such as those coding for the 16S rRNA molecule and the ribosomal protein S12, were not recognized. In the same sense, transferable trimethoprim-insensitive dihydrofolate reductase-encoding genes (*dfr*) (Bergmann et al., 2014) explaining the high MIC value of VA08-2AN to trimethoprim (8 μg mL^–1^) were not found. Further, the dihydropteroate synthase (DHPS) enzyme of *S. salivarius* was rather different to those of *Streptococcus pneumoniae* and *Streptococcus pyogenes* to search for substitutions causing resistance.

### Filter Mating Conjugation

Tetracycline-resistant strains containing one or two antibiotic resistance genes [*tet*(M) and/or *tet*(L)] were selected as donors for conjugation experiments. Under the assay conditions, neither of the two donors used, *E. faecalis* VA02-2 and *S. anginosus* VA01-10AN, was able to transfer resistance to tetracycline to any of the four recipient strains, with a detection limit < 10^–8^ transconjugants per donor cell.

### Cloning of *tet*(L) and Expression in *E. coli*

As a proof of concept of heterologous expression of the tetracycline resistance genes, the *tet*(L) gene from *S. anginosus* VA01-10AN was amplified with specific primers incorporating restriction enzyme sites for *Sal*I and *Eco*RI, ligated in double-digested pUC19, and electrotransformed in *E. coli*. Restriction enzyme analysis and sequencing of recombinant plasmids proved the inserted DNA to consist in the amplified *tet*(L) gene. *E*- test analysis of several *E. coli* transformants demonstrated the gene provided a tetracycline resistance of 48–64 μg mL^–1^ to this heterologous host.

## Discussion

This study examines the antibiotic resistance profiles of 15 isolates belonging to a phylogenetically related group of lactic acid-producing cocci from the human vagina. The isolates belonged to the species *E. faecalis* (9), *S. anginosus* (4), and *S. salivarius* (2). These numbers can be compared with the retrieval from the same samples of 25 lactic acid-producing rods (mostly lactobacilli) (Sirichoat et al., 2020). Strains of these cocci species are found in a variety of environments, including food, and human and animal mucosa. *E. faecalis* strains have been used as starters in food fermentations, and as human and animal probiotics (Nami et al., 2014; Ben Braïek and Smaoui, 2019). However, such uses are not without controversy due to the opportunistic pathogenicity of some strains, mostly in hospital settings, and the large antibiotic resistance carriage (Ben Braïek and Smaoui, 2019). The ability of the strains in this study to produce large amounts of lactic acid might assure the inhibition of most acid-susceptible vaginal pathogens (Matu et al., 2010).

In agreement with many other works in the literature (Huys et al., 2004; Hummel et al., 2007; Fernández-Fuentes et al., 2014; Kürekci et al., 2016; Chajêcka-Wierzchowska et al., 2019), all strains were susceptible to chloramphenicol, β-lactams (ampicillin and penicillin), vancomycin, linezolid, and trimethoprim. Enterococci are thought to be susceptible to vancomycin, and are considered intrinsically resistant to clindamycin, quinupristin-dalfopristin, cephalosporins and aminoglycosides (García-Solache and Rice, 2019). In agreement with this, the present enterococci strains proved to be quite resistant to all aminoglycosides (MIC ≥ 30 μg mL^–1^), with the exception of gentamicin. The MIC value of kanamycin in *S. salivarius* VA08-2AN was higher (128 μg mL^–1^) than in all other *Streptococcus* spp. isolates. However, genome analysis of this strain did not identify any gene or mutation known to be involved in kanamycin resistance. Unspecific mechanisms, including reduced uptake of the antibiotic by the lack of cytochrome-mediated transport or decreased cell permeability can lead to a concomitant resistance to kanamycin and other aminoglycosides (Pagès, 2017). Further, the situation of kanamycin resistance in *S. salivarius* can somehow be similar to that reported in anaerobic and facultative bacteria, where the presence of transferase-encoding genes, such as *aac*(6′)-*aph*(2′′), *ant*(6), *aph*(3′)-IIIa, *aph*(E) and *sat*(3), has not been unequivocally associated with resistance to this antibiotic (Ammor et al., 2008). Genome analysis of *E. faecalis* VA02-2 failed to identify a genetic cause for its low rifampicin resistance. Unspecific mechanisms, such as reduced permeability or reduced activity of efflux/influx mechanisms, have been suggested in other bacteria to be involved in low resistance to this antibiotic (Goldstein, 2014). Further, exceeding the MIC by one dilution falls within the normal variation for the microdilution assay (Huys et al., 2010).

Surprisingly, only five out of nine *E. faecalis* were resistant to clindamycin; these were also resistant to quinupristin-dalfopristin. The resistance of *E. faecalis* to clindamycin and quinupristin-dalfopristin is considered intrinsic and related to the activity of an ABC-F type efflux protein, LsaA, encoded by the *lsaA* gene (Singh et al., 2002; García-Solache and Rice, 2019). Sequence analysis of the amplicons from susceptible and resistant strains of this species showed the former to have mutations in the gene disrupting its ORF, leading to the production of non-functional proteins. This was corroborated by genome analysis of resistant and susceptible strains, including that of the reference strain *E. faecalis* OG1RF.

Enterococci and streptococci are mostly susceptible to tetracycline (García-Solache and Rice, 2019), although acquired resistance to this antibiotic has been reported widespread (Hummel et al., 2007; Cox and Wright, 2013; Fernández-Fuentes et al., 2014; Miller et al., 2014; Kürekci et al., 2016). PCR analyses identified *tet*(M) in all *E. faecalis* resistant strains, and both *tet*(M) and *tet*(L) in those of *S. anginosus* resistant to this antibiotic. The excretion of tetracycline as an active drug in urine could reasonably be a driver for the maintaining and spread of resistance genes in the vaginal ecosystem (Agwuh and MacGowan, 2006).

The location in *Enterococcus* and *Streptococcus* of AMR genes in transposons, integrons and ICEs is well known (Rizzotti et al., 2009; Roberts and Mullany, 2011; Mingoia et al., 2014; Zahid et al., 2017; León-Sampedro et al., 2019). The conjugative transference of *tet*(M) and *tet*(L) from different enterococci, such as *E. faecalis*, *E. faecium*, *Enterococcus mundtii* and *E. durans*, to enterococci and *Listeria* species has also been reported (Huys et al., 2004; Rizzotti et al., 2009). However, under the conditions of the present study, no transfer of *tet*(M) and/or *tet*(L) from *E. faecalis* or *S. anginosus* was seen. The Tn*916* transposon was found to be complete, but the sequence contained nucleotide changes and mutations that might affect its transfer to other bacteria. A substantial amount of data suggests that sub-inhibitory concentrations of antibiotics can increase the conjugation frequency of some genes (Lopatkin et al., 2016). However, in our study, a subinhibitory concentration of tetracycline during mating did not induce the conjugation process. ICEs, some of which carry tetracycline resistance genes, are composed of modules formed by genes or group of genes involved in maintenance and dissemination (Carraro and Burrus, 2014). Like Tn*916* in *E. faecalis* VA02-2, the ICE-like element found in *S. anginosus* VA01-10AN might be incomplete or non-functional. Nonetheless, spread of AMR genes can be accomplished by other processes such as transduction or natural transformation (Lerminiaux and Cameron, 2019). Moreover, *tet*(L) has been naturally found in more than 15 genera of Gram-positive and Gram-negative bacteria (Roberts and Schwarz, 2016). Expression of the *tet*(L) gene from *S. anginosus* in a heterologous host was, therefore, not surprising. This strongly suggests that if the gene is transferred to a bacterium by any means, it will likely be functional.

## Conclusion

In the vaginal ecosystem of the women involved in this study, only tetracycline resistance seems to have spread among *E. faecalis* and *S. anginosus*, which correlates with the presence of *tet*(M), or *tet*(L) plus *tet*(M), in the genomes of the resistant strains. In contrast, susceptibility to clindamycin and quinupristin-dalfopristin in some *E. faecalis* strains seemed to be due to disruptive mutations in *lsaA*. Neither *tet*(L) nor *tet*(M) from either the *E. faecalis* or *S. anginosus* strains was seen to be transferable under the assay conditions of this work. Nevertheless, *tet*(L) from *S. anginosu*s proved to be functional in a heterologous host, indicating that its carriage represents a hazard. This study provides new data on the antibiotic resistance/susceptibility profiles of vaginal *E. faecalis* and *Streptococcus* spp. strains and on the genetic basis of their intrinsic and acquired resistances.

## Data Availability Statement

The datasets presented in this study can be found in online repositories. The names of the repository/repositories and accession number(s) can be found at: https://www.ncbi.nlm.nih.gov/nuccore/?term=BioProject+PRJNA604905, BioPro-ject PRJNA604905.

## Ethics Statement

The study was approved by the Khon Kaen Ethics Committee in Human Research (Reference HE581191), and the sampling was performed following the standardized clinical guidelines of the Srinagarind Hospital (Khon Kaen). Written informed consent was obtained from each participant.

## Author Contributions

AS isolated the strains, performed the experimental work, and drafted the manuscript. AF and LV contributed to the antimicrobial testing and genome analyses. PB and MP contributed to planning the research and reviewed the drafts. VL and BM provided the financial and material resources, planned the experimental works, and reviewed the manuscript. All authors read and approved the final version.

## Conflict of Interest

The authors declare that the research was conducted in the absence of any commercial or financial relationships that could be construed as a potential conflict of interest.

## References

[B1] Agwuh K. N. MacGowan A. (2006). Pharmacokinetics and pharmacodynamics of the tetracyclines including glycylcyclines. *Antimicrob. Chemother.* 58 256–265. 10.1093/jac/dkl224 16816396

[B2] Altschul S. F. Madden T. L. Schäffer A. A. Zhang J. Zhang Z. Miller W. (1997). Gapped BLAST and PSI-BLAST: a new generation of protein database search programs. *Nucleic Acids Res.* 25 3389–3402. 10.1093/nar/25.17.3389 9254694 PMC146917

[B3] Ammor M. S. Flórez A. B. van Hoek A. H. A. de los Reyes-Gavilán C. G. Aarts H. J. M. Margolles A. (2008). Molecular characterization of intrinsic and acquired antibiotic resistance in lactic acid bacteria and bifidobacteria. *J. Mol. Microbiol. Biotechnol.* 14 6–15. 10.1159/000106077 17957105

[B4] Aziz R. K. Bartels D. Best A. A. DeJongh M. Disz T. Edwards R. A. (2008). The RAST Server: rapid annotations using subsystems technology. *BMC Genomics* 9:75. 10.1186/1471-2164-9-75 18261238 PMC2265698

[B5] Bankevich A. Nurk S. Antipov D. Gurevich A. A. Dvorkin M. Kulikov A. S. (2012). SPAdes: a new genome assembly algorithm and its applications to single-cell sequencing. *J. Comput. Biol.* 19 455–477. 10.1089/cmb.2012.0021 22506599 PMC3342519

[B6] Ben Braïek O. Smaoui S. (2019). Enterococci: between emerging pathogens and potential probiotics. *Biomed. Res. Int.* 2019:5938210. 10.1155/2019/5938210 31240218 PMC6556247

[B7] Bengtsson-Palme J. Kristiansson E. Larsson D. G. J. (2018). Environmental factors influencing the development and spread of antibiotic resistance. *FEMS Microbiol. Rev.* 42:fux053. 10.1093/femsre/fux053 29069382 PMC5812547

[B8] Bergmann R. van der Linden M. Chhatwal G. S. Nitsche-Schmitz D. P. (2014). Factors that cause trimethoprim resistance in *Streptococcus pyogenes*. *Antimicrob. Agents Chemother.* 58 2281–2288. 10.1128/AAC.02282-13 24492367 PMC4023743

[B9] Bolukaoto J. Y. Monyama C. M. Chukwu M. O. Lekala S. M. Nchabeleng M. Maloba M. R. (2015). Antibiotic resistance of *Streptococcus agalactiae* isolated from pregnant women in Garankuwa, South Africa. *BMC Res. Notes* 8:364. 10.1186/s13104-015-1328-0 26289147 PMC4544793

[B10] Carraro N. Burrus V. (2014). Biology of three ICE families: SXT/R391, ICE*Bs1*, and ICE*St1*/ICE*St3*. *Microbiol. Spectr.* 2:MDNA3-0008-2014. 10.1128/microbiolspec.MDNA3-0008-2014 26104437

[B11] Chajêcka-Wierzchowska W. Zadernowska A. Zarzecka U. Zakrzewski A. Gajewska J. (2019). Enterococci from ready-to-eat food - horizontal gene transfer of antibiotic resistance genes and genotypic characterization by PCR melting profile. *J. Sci. Food Agric.* 99 1172–1179. 10.1002/jsfa.9285 30047163

[B12] CLSI (2019). *Performance Standards for Antimicrobial Susceptibility Testing. Supplement M100*, 29th Edn Wayne, PA: Clinical and Laboratory Standards Institute.

[B13] Cox G. Wright G. D. (2013). Intrinsic antibiotic resistance: mechanisms, origins, challenges and solutions. *Int. J. Med. Microbiol.* 303 287–292. 10.1016/j.ijmm.2013.02.009 23499305

[B14] EFSA BIOHAZ Panel (2017). Scientific opinion on the update of the list of QPS-recommended biological agents intentionally added to food or feed as notified to EFSA. *EFSA J.* 15:e04664. 10.2903/j.efsa.2017.4664 32625421 PMC7010101

[B15] EFSA FEEDAP Panel (2012). Guidance on the assessment of bacterial susceptibility to antimicrobials of human and veterinary importance. *EFSA J.* 10:2740. 10.2903/j.efsa.2012.2740 29606757

[B16] EUCAST (2019). *The European Committee on Antimicrobial Susceptibility Testing. Breakpoint tables for interpretation of MICs and zone diameters. Version 9.0.* Available online at: http://www.eucast.org (accessed February 03, 2020).

[B17] FDA (2010). *Generally Recognized as Safe (GRAS). Notifications.* Available online at: https://www.fda.gov/food/generally-recognized-safe-gras/microorganisms-microbial-derived-ingredients-used-food-partial-list (accessed April 1, 2018).

[B18] Fernández-Fuentes M. A. Abriouel H. Ortega Morente E. Pérez Pulido R. Gálvez A. (2014). Genetic determinants of antimicrobial resistance in Gram positive bacteria from organic foods. *Int. J. Food Microbiol.* 172 49–56. 10.1016/j.ijfoodmicro.2013.11.032 24361832

[B19] Flórez A. B. Ammor M. S. Alvarez-Martín P. Margolles A. Mayo B. (2006). Molecular analysis of *tet*(W) gene-mediated tetracycline resistance in dominant intestinal *Bifidobacterium* species from healthy humans. *Appl. Environ. Microbiol.* 72 7377–7379. 10.1128/AEM.00486-06 16936047 PMC1636146

[B20] Flórez A. B. Ammor M. S. Mayo B. (2008). Identification of *tet*(M) in two *Lactococcus lactis* strains isolated from a Spanish traditional starter-free cheese made of raw milk and conjugative transfer of tetracycline resistance to lactococci and enterococci. *Int. J. Food Microbiol.* 121 189–194. 10.1016/j.ijfoodmicro.2007.11.029 18068255

[B21] Frank J. A. Reich C. I. Sharma S. Weisbaum J. S. Wilson B. A. Olsen G. J. (2008). Critical evaluation of two primers commonly used for amplification of bacterial 16S rRNA genes. *Appl. Environ. Microbiol.* 74 2461–2470. 10.1128/AEM.02272-07 18296538 PMC2293150

[B22] Fuochi V. Cardile V. Petronio Petronio G. Furneri P. M. (2019). Biological properties and production of bacteriocins-like-inhibitory substances by *Lactobacillus* sp. strains from human vagina. *J. Appl. Microbiol.* 126 1541–1550. 10.1111/jam.14164 30499608

[B23] García-Solache M. Rice L. B. (2019). The *Enterococcus*: a model of adaptability to its environment. *Clin. Microbiol. Rev.* 32:e00058-18. 10.1128/CMR.00058-18 30700430 PMC6431128

[B24] Goldstein B. P. (2014). Resistance to rifampicin: a review. *J. Antibiot. (Tokyo)* 67 625–630. 10.1038/ja.2014.107 25118103

[B25] Gupta S. K. Padmanabhan B. R. Diene S. M. Lopez-Rojas R. Kempf M. Landraud L. (2014). ARG-ANNOT, a new bioinformatic tool to discover antibiotic resistance genes in bacterial genomes. *Antimicrob. Agents Chemother.* 58 212–220. 10.1128/AAC.01310-13 24145532 PMC3910750

[B26] Huang B. Fettweis J. M. Brooks J. P. Jefferson K. K. Buck G. A. (2014). The changing landscape of the vaginal microbiome. *Clin. Lab. Med.* 34 747–761. 10.1016/j.cll.2014.08.006 25439274 PMC4254509

[B27] Hummel A. Holzapfel W. H. Franz C. M. (2007). Characterisation and transfer of antibiotic resistance genes from enterococci isolated from food. *Syst. Appl. Microbiol.* 30 1–7. 10.1016/j.syapm.2006.02.004 16563685

[B28] Huys G. D’Haene K. Cnockaert M. Tosi L. Danielsen M. Flórez A. B. (2010). Intra- and inter-laboratory performances of two commercial antimicrobial susceptibility testing methods for bifidobacteria and nonenterococcal lactic acid bacteria. *Antimicrob. Agents Chemother.* 54 2567–2574. 10.1128/AAC.00407-10 20385863 PMC2876413

[B29] Huys G. D’Haene K. Collard J. M. Swings J. (2004). Prevalence and molecular characterization of tetracycline resistance in *Enterococcus* isolates from food. *Appl. Environ. Microbiol.* 70 1555–1562. 10.1128/aem.70.3.1555-1562.2004 15006778 PMC368340

[B30] IDF (2010). *Milk and Milk Products : Determination of the Minimal Inhibitory Concentration (MIC) of Antibiotics Applicable to Bifidobacteria and non-Enterococcal Lactic acid Bacteria.* Brussels: International Dairy Federation.

[B31] Koeuth T. Versalovic J. Lupski J. R. (1995). Differential subsequence conservation of interspersed repetitive *Streptococcus pneumoniae* BOX elements in diverse bacteria. *Genome Res.* 5 408–418. 10.1101/gr.5.4.408 8750201

[B32] Krzyściak W. Pluskwa K. K. Jurczak A. Kościelniak D. (2013). The pathogenicity of the *Streptococcus* genus. *Eur. J. Clin. Microbiol. Infect. Dis.* 32 1361–1376. 10.1007/s10096-013-1914-9 24141975 PMC3824240

[B33] Kürekci C. Önen S. P. Yipel M. Aslanta” Ö Gündoðdu A. (2016). Characterisation of phenotypic and genotypic antibiotic resistance profile of enterococci from cheeses in Turkey. *Korean J. Food Sci. Anim. Resour.* 36 352–358. 10.5851/kosfa.2016.36.3.352 27433106 PMC4942550

[B34] León-Sampedro R. Fernández-de-Bobadilla M. D. San Millan A. Baquero F. Coque T. M. (2019). Transfer dynamics of Tn*6648*, a composite integrative conjugative element generated by tandem accretion of Tn*5801* and Tn*6647* in *Enterococcus faecalis*. *J. Antimicrob. Chemother.* 74 2517–2523. 10.1093/jac/dkz239 31225883

[B35] Lerminiaux N. A. Cameron A. D. S. (2019). Horizontal transfer of antibiotic resistance genes in clinical environments. *Can. J. Microbiol.* 65 34–44. 10.1139/cjm-2018-0275 30248271

[B36] Lopatkin A. J. Huang S. Smith R. P. Srimani J. K. Sysoeva T. A. Bewick S. (2016). Antibiotics as a selective driver for conjugation dynamics. *Nat. Microbiol.* 1:16044. 10.1038/nmicrobiol.2016.44 27572835 PMC5010019

[B37] Martin D. H. (2012). The microbiota of the vagina and its influence on women’s health and disease. *Am. J. Med. Sci.* 343 2–9. 10.1097/MAJ.0b013e31823ea228 22143133 PMC3248621

[B38] Martín R. Soberón N. Vaneechoutte M. Flórez A. B. Vázquez F. Suárez J. E. (2008). Characterization of indigenous vaginal lactobacilli from healthy women as probiotic candidates. *Int. Microbiol.* 11 261–266. 10.2436/20.1501.01.70 19204898

[B39] Mättö J. Malinen E. Suihko M. L. Alander M. Palva A. Saarela M. (2004). Genetic heterogeneity and functional properties of intestinal bifidobacteria. *J. Appl. Microbiol.* 97 459–470. 10.1111/j.1365-2672.2004.02340.x 15281925

[B40] Matu M. N. Orinda G. O. Njagi E. N. Cohen C. R. Bukusi E. A. (2010). *In vitro* inhibitory activity of human vaginal lactobacilli against pathogenic bacteria associated with bacterial vaginosis in Kenyan women. *Anaerobe* 16 210–215. 10.1016/j.anaerobe.2009.11.002 19925874

[B41] McArthur A. G. Waglechner N. Nizam F. Yan A. Azad M. A. Baylay A. J. (2013). The comprehensive antibiotic resistance database. *Antimicrob. Agents Chemother.* 57 3348–3357. 10.1128/AAC.00419-13 23650175 PMC3697360

[B42] Miller W. R. Munita J. M. Arias C. A. (2014). Mechanisms of antibiotic resistance in enterococci. *Expert. Rev. Anti. Infect. Ther.* 12 1221–1236. 10.1586/14787210.2014.956092 25199988 PMC4433168

[B43] Mingoia M. Morici E. Morroni G. Giovanetti E. Del Grosso M. Pantosti A. (2014). Tn*5253* family integrative and conjugative elements carrying *mef*(I) and *catQ* determinants in *Streptococcus pneumoniae* and *Streptococcus pyogenes*. *Antimicrob. Agents Chemother.* 58 5886–5893. 10.1128/AAC.03638-14 25070090 PMC4187955

[B44] Nagaraja P. (2008). Antibiotic resistance of *Gardnerella vaginalis* in recurrent bacterial vaginosis. *Indian J. Med. Microbiol.* 26 155–157. 10.4103/0255-0857.40531 18445953

[B45] Nami Y. Abdullah N. Haghshenas B. Radiah D. Rosli R. Yari Khosroushahi A. (2014). A newly isolated probiotic *Enterococcus faecalis* strain from vagina microbiota enhances apoptosis of human cancer cells. *J. Appl. Microbiol.* 117 498–508. 10.1111/jam.12531 24775273

[B46] Nogacka A. Salazar N. Suárez M. Milani C. Arboleya S. Solís G. (2017). Impact of intrapartum antimicrobial prophylaxis upon the intestinal microbiota and the prevalence of antibiotic resistance genes in vaginally delivered full-term neonates. *Microbiome* 5:93. 10.1186/s40168-017-0313-3 28789705 PMC5549288

[B47] Pagès J. M. (2017). Antibiotic transport and membrane permeability: new insights to fight bacterial resistance. *Biol. Aujourdhui* 211 149–154. 10.1051/jbio/2017020 29236663

[B48] Peterson E. Kaur P. (2018). Antibiotic resistance mechanisms in bacteria: relationships between resistance determinants of antibiotic producers, environmental bacteria, and clinical pathogens. *Front. Microbiol.* 9:2928. 10.3389/fmicb.2018.02928 30555448 PMC6283892

[B49] Rizzotti L. La Gioia F. Dellaglio F. Torriani S. (2009). Molecular diversity and transferability of the tetracycline resistance gene *tet*(M), carried on Tn*916*-*1545* family transposons, in enterococci from a total food chain. *Antonie Van Leeuwenhoek* 96 43–52. 10.1007/s10482-009-9334-7 19333776

[B50] Roberts A. P. Mullany P. (2011). Tn*916*-like genetic elements: a diverse group of modular mobile elements conferring antibiotic resistance. *FEMS Microbiol. Rev.* 35 856–871. 10.1111/j.1574-6976.2011.00283.x 21658082

[B51] Roberts M. C. Schwarz S. (2016). Tetracycline and phenicol resistance genes and mechanisms: importance for agriculture, the environment, and humans. *J. Environ. Qual.* 45 576–592. 10.2134/jeq2015.04.0207 27065405

[B52] Rossetti L. Giraffa G. (2005). Rapid identification of dairy lactic acid bacteria by M13-generated, RAPD-PCR fingerprint databases. *J. Microbiol. Methods* 63 135–144. 10.1016/j.mimet.2005.03.001 15893395

[B53] Sambrook J. Russell D. W. (2001). *Molecular Cloning: a Laboratory Manual.* New York, NY: Cold Spring Harbor Laboratory Press.

[B54] Sansevere E. A. Robinson D. A. (2017). Staphylococci on ICE: overlooked agents of horizontal gene transfer. *Mob. Genet. Elements* 7 1–10. 10.1080/2159256X.2017.1368433 28932624 PMC5599092

[B55] Singh K. V. Weinstock G. M. Murray B. E. (2002). An *Enterococcus faecalis* ABC homologue (Lsa) is required for the resistance of this species to clindamycin and quinupristin-dalfopristin. *Antimicrob. Agents Chemother.* 46 1845–1850. 10.1128/aac.46.6.1845-1850.2002 12019099 PMC127256

[B56] Sirichoat A. Buppasiri P. Engchanil C. Namwat W. Faksri K. Sankuntaw N. (2018). Characterization of vaginal microbiota in Thai women. *PeerJ* 6:e5977. 10.7717/peerj.5977 30498641 PMC6252066

[B57] Sirichoat A. Flórez A. B. Vázquez L. Buppasiri P. Panya M. Lulitanond V. (2020). Antibiotic susceptibility profiles of lactic acid bacteria from the human vagina and genetic basis of acquired resistances. *Int. J. Mol. Sci.* 21:2594. 10.3390/ijms21072594 32276519 PMC7178285

[B58] Smith S. B. Ravel J. (2017). The vaginal microbiota, host defence and reproductive physiology. *J. Physiol.* 595 451–463. 10.1113/JP271694 27373840 PMC5233653

[B59] Tatusova T. DiCuccio M. Badretdin A. Chetvernin V. Nawrocki E. P. Zaslavsky L. (2016). NCBI prokaryotic genome annotation pipeline. *Nucleic Acids Res.* 44 6614–6624. 10.1093/nar/gkw569 27342282 PMC5001611

[B60] van Hoek A. H. Mevius D. Guerra B. Mullany P. Roberts A. P. Aarts H. J. (2011). Acquired antibiotic resistance genes: an overview. *Front. Microbiol.* 2:203. 10.3389/fmicb.2011.00203 22046172 PMC3202223

[B61] Zahid S. Bin-Asif H. Hasan K. A. Rehman M. Ali S. A. (2017). Prevalence and genetic profiling of tetracycline resistance (*Tet-R*) genes and transposable element (Tn*916*) in environmental *Enterococcus* species. *Microb. Pathog.* 111 252–261. 10.1016/j.micpath.2017.09.009 28888881

[B62] Zankari E. Hasman H. Cosentino S. Vestergaard M. Rasmussen S. Lund O. (2012). Identification of acquired antimicrobial resistance genes. *J. Antimicrob. Chemother.* 67 2640–2644. 10.1093/jac/dks261 22782487 PMC3468078

