# Supplementary material for: Antibiotic Resistance-Susceptibility Profiles of Enterococcus faecalis and Streptococcus spp. From the Human Vagina, and Genome Analysis of the Genetic Basis of Intrinsic and Acquired Resistances
Source: Front Microbiol. 2020 Jun 26;11:1438. doi: 10.3389/fmicb.2020.01438 (PMC7333779; doi:10.3389/fmicb.2020.01438)
Supplement: Supplementary file 1 [file Data_Sheet_1.PDF]

Supplementary Table 1.- Primers and PCR conditions for amplifying selected antibiotic resistance genes used in this study.

Resistance gene	Primers	Sequence (5' – 3')	*Tm* (ºC)	Amplicon size (bp)	Reference
RPP[a]	DI<br>DII	GAYACNCCNGGNCAYRTNGAYTT<br>GCCCARWANGGRTTNGGNGGNACYTC	50	1,083	Clermont et al. (1997)
RPP	Tet-1<br>Tet-2	GCTCACGTTGACGCAGGAA<br>AGGATTTGGCGGGACTTCTA	50	1,300	Barbosa et al. (1999)
tet(M)	DI<br>tetM_R	GAYCANCCNGGNCAYRTNGAYTT<br>CACCGAGCAGGGATTTCTCCAC	55	1,513	Gevers et al. (2003)
tet(O)	tetO_F<br>tetO_R	AATGAAGATTCCGACAATTT<br>CTCATGCGTTGTAGTATTCCA	45	781	Gevers et al. (2003)
tet(S)	tetS_F<br>tetS_R	ATCAAGATATTAAGGAC<br>TTCTCTATGTGGTAATC	45	573	Gevers et al. (2003)
tet(W)	tetWF<br>Tet-2	AAGCGGCAGTCACTTCCTTCC<br>AGGATTTGGCGGGACTTCTA	50	1,200	Scott et al. (2000)
tet(K)	tetK_F<br>tetK_R	TTATGGTGGTTGTAGCTAGAAA<br>AAAGGGTTAGAAACTCTTGAAA	45	348	Gevers et al. (2003)
tet(L)	tetL_F<br>tetL_R	GTMGTTGCGCGCTATATTCC<br>GTGAAMGRWAGCCCACCTAA	45	696	Gevers et al. (2003)
erm(A)	ermA_F<br>ermA_R	TCTAAAAAGCATGTAAAAGAA<br>CTTCGATAGTTTATTAATATTAGT	48	645	Rizzotti et al. (2005)
erm(B)	ermB_F<br>ermB_R	GAAAAGGTACTCAACCAAATA<br>AGTAACGGTACTTAAATTGTTTAC	50	639	Rizzotti et al. (2005)
erm(C)	ermC_F<br>ermC_R	TCAAAACATAATATAGATAAA<br>GCTAATATTGTTTAAATCGTCAAT	43	642	Rizzotti et al. (2005)
erm(F)	ermF_F<br>ermF_R	CGGGTCAGCACTTTACTATTG<br>GGACCTACCTCATAGACAAG	50	466	Roberts et al. (1999)
mef(A)	mefA_F<br>mefA_R	ACCGATTCTATCAGCAAAG<br>GGACCTGCCATTGGTGTG	43	940	Luna et al. (2000)
cat	cat_F<br>cat_R	ATGACTTTTAATATTATTRAWTT<br>TCATYTACMYTATSAATTATAT	49	648	Hummel et al. (2007a)
bla	bla_F<br>bla_R	CATARTTCCGATAATASMGCC<br>CGTSTTTAACTAAGTATSGY	51	297	Hummel et al. (2007b)
aac(6')-aph(2")	aac_F<br>aac_R	CCAAGAGCAATAAGGGCATA<br>CACTATCATAACCACTACCG	60	220	Rojo-Bezares et al. (2006)
aad(E)	aadE_F<br>aadE_R	GCAGAACAGGATGAACGTATTCG<br>ATCAGTCGGAACTATGTCCC	55	369	Klare et al. (2007)
lsa	lsa_F<br>lsa_R	GTAAAGCTGCATCAATTTTGC<br>CATGAAATGTGCATCATGCTC	50	1,496	This study
*van*A	vanA_F<br>vanA_R	TCACCCCTTTAACGCTAATAC<br>GTTTGGGGGTTGCTCAGACC	50	1,006	This study
tet(L)[b]	tetL-F-Sal<br>tetL-R-Eco	CGTCGACCAAATAGTCGGATAGATAAAGTACG<br>CTTGAATTCAGCAATCGCGCCCTTTAATGGAAC	68	1,627	This study

[a]RPP, genes encoding ribosomal protecting proteins.
[b]These primers amplify the gene and its promoter and terminator regions; the sequences of the restriction enzyme sites incorporated to the primers are underlined.

References

- Barbosa, T.M., Scott, K.P., and Flint, H.J. (1999). Evidence for recent intergeneric transfer of a new tetracycline resistance gene, *tet*(W), isolated from *Butyrivibrio fibrisolvens*, and the occurrence of *tet*(O) in ruminal bacteria. *Environ Microbiol* 1: 53-64.

- Clermont, D., Chesneau, O., De Cespedes, G., and Horaud, T. (1997). New tetracycline resistance determinants coding for ribosomal protection in streptococci and nucleotide sequence of *tet*(T) isolated from *Streptococcus pyogenes* A498. *Antimicrob Agents Chemother* 41: 112-6.

- Gevers, D., Danielsen, M., Huys, G., and Swings, J. (2003). Molecular characterization of *tet*(M) genes in *Lactobacillus* isolates from different types of fermented dry sausage. *Appl Environ Microbiol* 69: 1270-5.

- Hummel, A., Holzapfel, W.H., and Franz, C.M. (2007a). Characterisation and transfer of antibiotic resistance genes from enterococci isolated from food. *Syst Appl Microbiol* 30(1), 1-7. doi: 10.1016/j.syapm.2006.02.004.

- Hummel, A.S., Hertel, C., Holzapfel, W.H., and Franz, C.M. (2007b). Antibiotic resistances of starter and probiotic strains of lactic acid bacteria. *Appl Environ Microbiol* 73(3), 730-739. doi: 10.1128/AEM.02105-06.

- Klare, I., Konstabel, C., Werner, G., Huys, G., Vankerckhoven, V., Kahlmeter, G., Hildebrandt, B., Müller-Bertling, S., Witte, W., and Goossens, H. (2007). Antimicrobial susceptibilities of *Lactobacillus, Pediococcus* and *Lactococcus* human isolates and cultures intended for probiotic or nutritional use. *J Antimicrob Chemother* 59(5), 900-912. doi: 10.1093/jac/dkm035.

- Luna, V.A., Cousin, S., Jr., Whittington, W.L., and Roberts, M.C. (2000). Identification of the conjugative mef gene in clinical *Acinetobacter junii* and *Neisseria gonorrhoeae* isolates. *Antimicrob Agents Chemother* 44: 2503-6.

- Rizzotti, L., Simeoni, D., Cocconcelli, P., Gazzola, S., Dellaglio, F., and Torriani, S. (2005). Contribution of enterococci to the spread of antibiotic resistance in the production chain of swine meat commodities. *J Food Prot* 68: 955-65.

- Roberts, M.C., Chung, W.O., Roe, D., Xia, M., Marquez, C., Borthagaray, G., Whittington, W.L., and Holmes, K.K. (1999). Erythromycin-resistant *Neisseria gonorrhoeae* and oral commensal *Neisseria* spp. carry known rRNA methylase genes. *Antimicrob Agents Chemother* 43: 1367-72.

- Rojo-Bezares, B., Saenz, Y., Poeta, P., Zarazaga, M., Ruiz-Larrea, F., and Torres, C. (2006). Assessment of antibiotic susceptibility within lactic acid bacteria strains isolated from wine. *Int J Food Microbiol* 111: 234-40.

- Scott, K.P., Melville, C.M., Barbosa, T.M., and Flint, H.J. (2000). Occurrence of the new tetracycline resistance gene *tet*(W) in bacteria from the human gut. *Antimicrob Agents Chemother* 44: 775-7.

Supplementary Table 2.- Analysis of open reading frames (ORFs) in the contigs from the genome of *Enterococcus faecalis* VA02-2 (179,144 bp) and *Streptococcus anginosus* VA01-14AN (95,740 bp) strains around the position harbouring the tetracycline resistant genes *tet*(M), and *tet*(M) and *tet*(L), respectively.

ORF	5' end position	3' end position[a]	% GC content	No. of aa[b]	Known protein with the highest homology (microorganism)[c]	Identity length/total length (% aa identity)	GenBank accession no.
***E. faecalis* VA02-2**							
ORF1	20,534	22,357	39.5	607	APC family permease (Bacilli)	607/607 (100%)	WP_002379167.1
ORF2	22,858	22,394	39.6	154	GNAT family N-acetyltransferase (*Enterococcus faecalis*)	154/154 (100%)	WP_002425408.1
ORF3	23,592	22,930	39.4	220	ABC transporter permease (Bacilli)	220/220 (100%)	WP_002363261.1
ORF4	24,519	23,596	37.6	307	Osmoprotectant ABC transporter substrate-binding protein (Bacilli)	307/307 (100%)	WP_002363262.1
ORF5	25,154	24,519	38.2	211	ABC transporter permease (Bacilli)	211/211 (100%)	WP_002355744.1
ORF6	26,342	25,158	37.6	394	Betaine/proline/choline family ABC transporter ATP-binding protein (*E. faecalis*)	393/397 (99%)	WP_141417045.1
ORF7	26,772	27,086	40.3	104	YdcP family protein, partial (*Streptococcus agalactiae*)	104/119 (100%)	WP_000313304.1
ORF8	27,105	27,488	41.7	127	YdcP family protein (Bacteria)	127/128 (100%)	WP_000985015.1
ORF9	27,517	28,902	40.0	461	DNA translocase FtsK (Bacteria)	461/461 (100%)	WP_000813488.1
ORF10	29,080	30,285	41.1	401	XRE family transcriptional regulator (*Streptococcus pneumoniae*)	401/472 (100%)	VJU37263.1
ORF11	30,328	30,549	37.4	73	Conjugation related protein (*S. pneumoniae*)	73/82 (100%)	VMD55525.1
ORF12	30,666	31,163	40.4	165	Antirestriction protein ArdA (Bacteria)	165/165 (100%)	WP_000342539.1
ORF13	31,252	31,644	39.7	130	Conjugal transfer protein (Firmicutes)	130/130 (100%)	WP_000723888.1
ORF14	31,628	34,075	40.7	815	ATP-binding protein (*Streptococcus suis*)	815/833 (100%)	ABP89892.1
ORF15	34,078	36,255	41.7	725	YtxH domain-containing membrane protein (*E. faecalis*)	725/725 (100%)	AAB60018.1
ORF16	36,252	37,253	42.7	333	Peptidase P60; glycoside hydrolase family 23 (*S. pneumoniae*)	333/338 (100%)	WP_050278213.1
ORF17	37,250	38,182	37.4	310	Conjugal transfer protein (Bacteria)	310/310 (100%)	WP_001224318.1
ORF18	38,559	40,478	36.1	639	Tetracycline resistance ribosomal protection protein Tet(M) (Terrabacteria group)	639/639 (100%)	WP_000691736.1
ORF19	41,177	40,824	36.4	117	Helix-turn-helix transcriptional regulator (Bacteria)	117/117 (100%)	WP_001227347.1
ORF20	41,682	42,104	37.6	140	Sigma-70 family RNA polymerase sigma factor (Bacteria)	140/140 (100%)	WP_000804885.1
ORF21	42,101	42,331	37.2	76	Helix-turn-helix domain-containing protein (Bacteria)	76/76 (100%)	WP_000857133.1
ORF22	42,523	42,395	36.4	42	Hypothetical protein (*Mageeibacillus indolicus*)	42/42 (100%)	ADC90905.1
ORF23	42,792	42,995	35.3	67	Chain A, excisionase from transposon *Tn*916 (*E. faecalis*)	67/70 (100%)	1Y6U_A
ORF24	43,076	44,293	37.3	405	Transposase (Bacteria)	405/405 (100%)	WP_001291561.1
ORF25	45,906	45,016	38.9	296	Glyoxalase, ring-cleaving dioxygenase (*E. faecalis*)	296/296 (100%)	WP_010822602.1
ORF26	46,072	47,103	38.8	343	tRNA preQ1(34) S-adenosylmethionine ribosyltransferase-	343/343 (100%)	WP_141417044.1

					isomerase QueA (*E. faecalis*)		
ORF27	47,696	47,448	39.8	82	Helix-turn-helix transcriptional regulator (*Enterococcus*)	82/82 (100%)	WP_002381292.1
ORF28	48,577	51,222	39.7	881	Calcium-translocating P-type ATPase, PMCA-type (*E. faecalis*)	881/881 (100%)	WP_141417043.1
ORF29	51,556	53,562	37.6	668	Potassium transporter Kup (Bacilli)	668/668 (100%)	WP_002381294.1
ORF30	54,330	53,632	34.3	232	Helix-turn-helix domain-containing protein (*E. faecalis*)	232/232 (100%)	WP_141417042.1
ORF31	54,495	56,600	38.3	701	Copper-translocating P-type ATPase (*E. faecalis*)	701/701 (100%)	WP_141417041.1
ORF32	56,724	58,148	33.0	474	M protein trans-acting positive regulator (*E. faecalis*)	474/474 (100%)	WP_141417040.1
ORF33	58,217	59,128	37.3	303	Aldo/keto reductase (*E. faecalis*)	303/303 (100%)	WP_010716825.1
***S. anginosus* VA01-10AN**							
ORF1	55,041	56,072	33.2	343	Conjugative transposon protein (*Streptococcus agalactiae*)	343/360 (100%)	EPV08632.1
ORF2	56,154	63,431	37.8	2,425	Hypothetical protein (*Streptococcus*)	2424/2425 (99%)	WP_024051476.1
ORF3	63,531	63,737	32.4	68	Helix-turn-helix transcriptional regulator (*Streptococcus*)	68/68 (100%)	WP_024051477.1
ORF4	63,738	64,859	26.4	373	Dam family site-specific DNA-(adenine-N6)-methyltransferase (*Streptococcus*)	373/373 (100%)	WP_070654359.1
ORF5	64,864	65,637	30.7	257	Hypothetical protein (*Streptococcus anginosus*)	257/257 (100%)	ETI87161.1
ORF6	65,860	66,150	25.1	96	DUF3847 domain-containing protein (*Streptococcus*)	96/96 (100%)	WP_024051480.1
ORF7	66,495	68,135	27.3	546	Nickase (*S. anginosus*)	546/546 (100%)	WP_150867877.1
ORF8	68,209	68,382	28.7	57	Bacterial nucleoid DNA-binding protein (*S. anginosus*)	57/57 (100%)	ETI87079.1
ORF9	68,556	69,077	23.8	173	Hypothetical protein (*Streptococcus*)	173/173 (100%)	WP_024051516.1
ORF10	69,284	69,811	29.2	175	PcfB family protein (*S. anginosus*)	175/175 (100%)	WP_101800919.1
ORF11	69,937	70,671	31.2	244	Replisome organizer (*S. anginosus*)	244/244 (100%)	WP_101800920.1
ORF12	70,829	70,990	30.2	53	Hypothetical protein (Bacteria)	53/53 (100%)	WP_019213949.1
ORF13	71,069	72,688	30.9	539	DUF4368 domain-containing protein (*Streptococcus*)	539/539 (100%)	WP_024052578.1
ORF14	72,685	74,202	38.1	505	Hypothetical protein (*Streptococcus*)	505/505 (100%)	WP_024052577.1
ORF15	74,243	74,902	36.2	219	Hypothetical protein (Firmicutes)	219/219 (100%)	WP_000384996.1
ORF16	76,302	74,971	36.9	443	Relaxase/mobilization nuclease domain-containing protein (Firmicutes)	443/443 (100%)	WP_001018662.1
ORF17	76,661	76,305	34.2	118	Plasmid mobilization relaxosome protein MobC (Firmicutes)	118/118 (100%)	WP_000013547.1
ORF18	76,970	77,443	27.6	157	Transcriptional regulator, TetR family (*S. anginosus*)	157/157 (100%)	ETI84793.1
ORF19	77,784	78,098	40.3	104	YdcP family protein, partial (*S. agalactiae*)	104/119 (100%)	WP_000313304.1
ORF20	78,117	78,500	41.7	127	YdcP family protein (Bacteria)	127/128 (100%)	WP_000985015.1
ORF21	78,529	79,914	40.0	461	DNA translocase FtsK (Bacteria)	461/461 (100%)	WP_000813488.1
ORF22	80,092	81,297	41.3	401	Transcriptional regulator protein (*S. pneumoniae*)	401/458 (100%)	VJU37263.1
ORF23	81,340	81,561	37.4	73	Conjugation related protein (*S. pneumoniae*)	73/82 (100%)	VMD55525.1
ORF24	81,678	82,175	40.4	165	Antirestriction protein ArdA (Bacteria)	165/165 (100%)	WP_000342539.1
ORF25	82,264	82,656	39.7	130	Conjugal transfer protein (Bacteria)	130/130 (100%)	WP_000506270.1
ORF26	82,640	85,087	40.7	815	Hypothetical protein (*S. suis*)	815/818 (100%)	ABP89892.1

ORF27	85,090	87,267	41.7	725	Membrane protein (*S. agalactiae*)	725/725 (100%)	WP_029763131.1
ORF28	87,264	88,265	42.7	333	Peptidase P60 (*S. pneumoniae*)	333/338 (100%)	WP_050278213.1
ORF29	88,262	89,197	37.5	311	Conjugal transfer protein (*Streptococcus*)	310/311 (99%)	WP_070672379.1
ORF30	89,574	91,493	37.2	639	Tetracycline resistance ribosomal protection protein Tet(M) (*Streptococcus*)	639/639 (100%)	WP_070654311.1
ORF31	91,591	92,964	35.3	457	Tetracycline efflux MFS transporter Tet(L) (Lactobacillales)	457/457 (100%)	WP_002345004.1
ORF32	93,528	94,790	33.8	420	Plasmid recombination enzyme (*Staphylococcus sciuri*)	420/446 (100%)	AQM75261.1
ΔORF33	95,035	95,634	33.2	199	Plasmid RC replicating protein, partial (*Staphylococcus aureus*)	193/197 (100%)	WP_001795117.1

[a]Including start and stop codons.

[b]aa, amino acids.

[c]Color code of the different open reading frames (ORFs): Purple, antibiotic resistance genes; red, genes involved in plasmid replication and control; green, transposase-, integrase-, mobilization, and conjugation-associated genes; orange, genes encoding transcription regulators; white, genes involved in other processes. The color code is the same as in Figure 1.

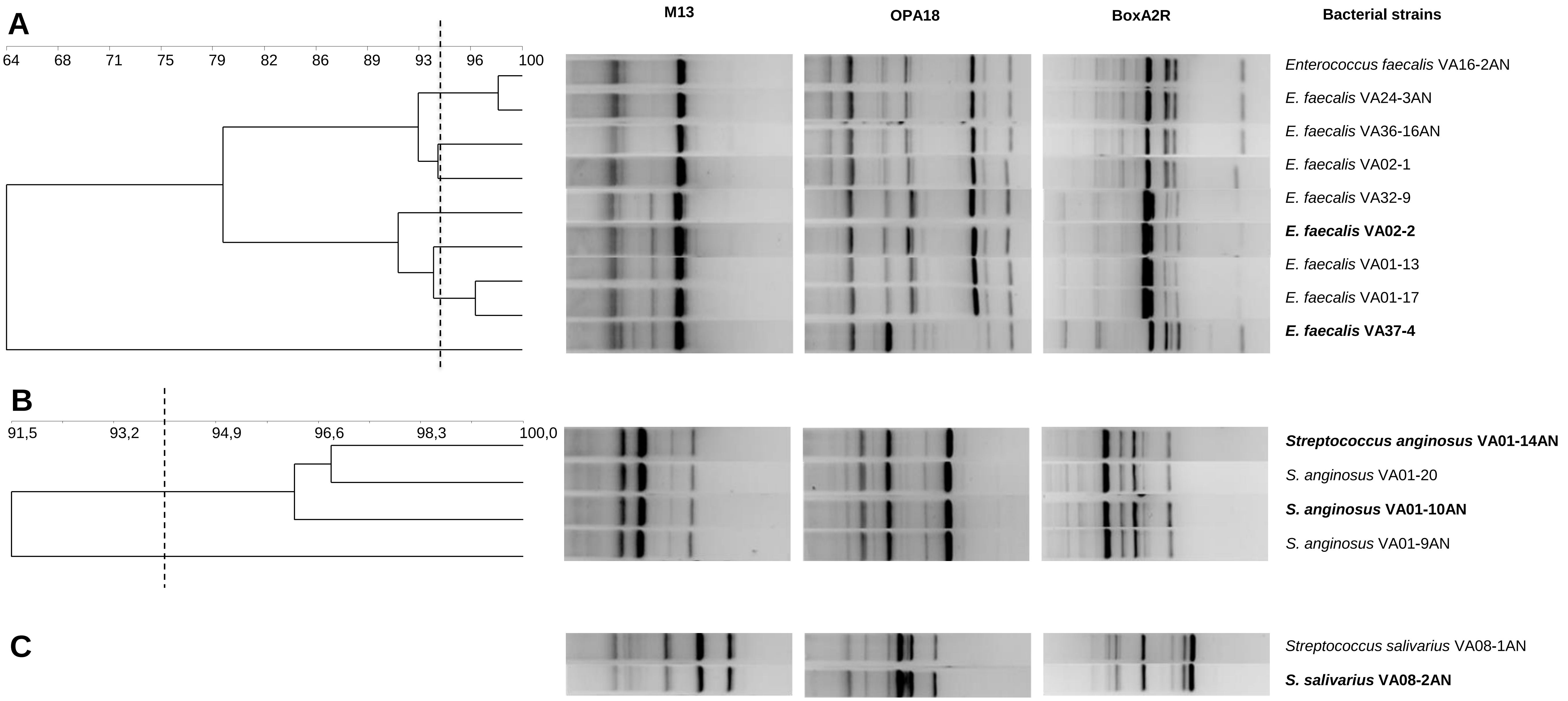

Supplementary Figure 1.- Representative rep-PCR and RAPD typing profiles of the strains of *Enterococcus faecalis* (A) , *Streptococcus anginosus* (B) and *Streptococcus salivarius* (C) obtained with primers M13, OPA18 and BoxA2R. On the left, dendrogram of similarity of the combined typing profiles expressed by the Simple Matching (SM) coefficient. Clustering was performed by the unweighted pair group method using arithmetic averages (UPGMA). The vertical dotted lines designate the similarity level (94%; minimum level of reproducibility) chosen as discriminatory at the strain level. The strains in bold were selected for genome sequencing.

```
                                   10         20         30         40         50
OG1RF   [CLI^r/QDA^r]     MSKIELKQLS FAYDNQEVLL FDQANITMDT NWKLGLIGRN GRGKTTLLRL
VA37-4  [CLI^r/QDA^r]     ---------- ---------- ---------- ---------- ----------
VA02-2  [CLI^s/QDA^s]     ---------- ---------- ---------- ---------- ----------

                                   60         70         80         90        100
OG1RF   [CLI^r/QDA^r]     LQKQLDYQGE ILHQVDFVYF PQTVAEEQQL TYYVLQEVTS FEQWELEREL
VA37-4  [CLI^r/QDA^r]     ---------- ---------- ------G--- ---------- ----------
VA02-2  [CLI^s/QDA^s]     ---------- ---------- ---------- ---------- ----K-----

                                  110        120        130        140        150
OG1RF   [CLI^r/QDA^r]     TLLNVDPEVL WRPFSSLSGG EKTKVLLGLL FIEENAFPLI DEPTNHLDLA
VA37-4  [CLI^r/QDA^r]     ---------- ---------- ---------- ---------- ----------
VA02-2  [CLI^s/QDA^s]     ---------- ---------- ---------- ---------- ----------

                                  160        170        180        190        200
OG1RF   [CLI^r/QDA^r]     GRQQVAEYLK KKKHGFILVS HDRAFVDEVV DHILAIEKSQ LTLYQGNFSI
VA37-4  [CLI^r/QDA^r]     ---------- ---------- ---------- ---------- ----------
VA02-2  [CLI^s/QDA^s]     --*

                                  210        220        230        240        250
OG1RF   [CLI^r/QDA^r]     YEEQKKLRDA FELAENEKIK KEVNRLKETA RKKAEWSMNR EGDKYGNAKE
VA37-4  [CLI^r/QDA^r]     ---------- ---------- ---------- ---------- ----------
VA02-2  [CLI^s/QDA^s]

                                  260        270        280        290        300
OG1RF   [CLI^r/QDA^r]     KGSGAIFDTG AIGARAARVM KRSKHIQQRA ETQLAEKEKL LKDLEYIDPL
VA37-4  [CLI^r/QDA^r]     ---------- ---------- ---------- ---------- ----------
VA02-2  [CLI^s/QDA^s]

                                  310        320        330        340        350
OG1RF   [CLI^r/QDA^r]     SMDYQPTHHK TLLTVEELRL GYEKNWLFAP LSFSINAGEI VGITGKNGSG
VA37-4  [CLI^r/QDA^r]     ---------- ---------- ---------- I--------- ----------
VA02-2  [CLI^s/QDA^s]

                                  360        370        380        390        400
OG1RF                     KSSLIQYLLD NFSGDSEGEA TLAHQLTISY VRQDYEDNQG TLSEFAEKNQ
VA37-4  [CLI^r/QDA^r]     ---------- ---------- ---------- ---------- ----------
VA02-2  [CLI^s/QDA^s]                   *

                                  410        420        430        440        450
OG1RF   [CLI^r/QDA^r]     LDYTQFLNNL RKLGMERAVF TNRIEQMSMG QRKKVEVAKS LSQSAELYIW
VA37-4  [CLI^r/QDA^r]     ---------- ---------- ---------- ---------- ----------
VA02-2  [CLI^s/QDA^s]

                                  460        470        480        490      498
OG1RF   [CLI^r/QDA^r]     DEPLNYLDVF NHQQLEALIL SVKPAMLVIE HDAHFMKKIT DKKIVLKS
VA37-4  [CLI^r/QDA^r]     ---------- ---------- ---------- ---------- ----A---
VA02-2  [CLI^s/QDA^s]
```

Supplementary Figure 2.- Alignment of the deduced amino acid sequence of LsaA ABC-F family type ribosomal protection protein encoded by *lsaA* genes from clindamycin and quinupristin-dalfopristin resistant and susceptible *E. faecalis* strains. As a reference, the sequence of LsaA from the clindamycin and quinupristin-dalfopristin resistant strain *E. faecalis* OG1RF (CP002621.1) was used. Amino acid changes as compared to the control strain are color highlighted. The asterisks (*) denoted positions where a stop codon is introduced in the tetracycline resistant [tet(M)], clindamicin and quinupristin-dalfopristin susceptible strain *E. faecalis* VA02.2.
